# Supplementary material for: Bayesian rank likelihood-based estimation: An application to low birth weight in Ethiopia
Source: PLoS One. 2024 May 31;19(5):e0303637. doi: 10.1371/journal.pone.0303637 (PMC11142591; doi:10.1371/journal.pone.0303637)
Supplement: Attachment — Submitted filename: Response to reviewers.pdf [file pone.0303637.s002.pdf]

Responses to the academic editor and reviewers' comments

First of all, we would like to say thank you for your constructive and very important comments. We accepted the comments and made appropriate changes.

Response to the academic editor

- If you consider the Bayesian model as a novel (applied since 1976), as raised by Reviewer #2, you have to do a methodological comparison with conventional models.
    - ✓ We conducted a methodological comparison based on the feedback provided by the reviewer and academic editor.
- I could see that labels for your convergence algorism assessment are missing and confusing for readers. Besides, a few of your plots are hard to visualize if converged or not.
    - ✓ We explained what those plots indicate in the paper's results section.
- Additionally, it is not clear which form of Bayesian you applied in your analysis (informative, non-informative).
    - ✓ We used both informative and non-informative priors for the model parameters in our paper on page 5.
- Major concern, your discussion is too shallow and the possible explanations are not plausible. Please also include implications of your study for policy or other context.
    - ✓ We took the comments seriously and made an effort to incorporate them as best we could.

Response to Reviewer 1

- Overall, a thorough review of the manuscript for grammatical issues is still required. Even within the abstract and introduction, multiple grammatical issues make it difficult to read. Editing by a colleague or writing service with strong English skills is needed.
    - ✓ We have sent the paper to a colleague for language editing.
- It would be better if the abstract could be structured as follows: background, methods, results, and conclusion.
    - ✓ We wrote the abstract according to the comment
- As a justification for the study, you stated, "Most studies conducted in Ethiopia have utilized standard models employing maximum likelihood estimation with small sample sizes." However, it could be better if you describe the limitations of those

standard models and the advantage of Bayesian rank likelihood-based estimation over the standard models.

  - ✓ We described the limitations of those standard models in the abstract as per the comment

- The abstract should explain at least the data source, the study population, and the sample size.
  - ✓ The data source, study population and sample size are explained in the abstract accordingly

- The abstract should be a stand-alone document. Some of your statements in the abstract are not standalone. For example, you stated, “The prevalence of low birth weight varies across different regions of the country, highlighting the need for targeted interventions in areas with a higher prevalence." This may need further clarification where the prevalence is high.
  - ✓ We tried to address the comment as per the comment
- Overall, in the abstract, I couldn’t find the answer to your study objective: to estimate parameters and provide a nationwide estimation of low birth weight and its risk factors in Ethiopia. At least you have to present the magnitude of LBW according to your estimation.
  - ✓ We tried to address the comment as per the comment
- The study revealed significant associations between birth weight and the mother's age, number of antenatal care visits, birth order, and BMI. What is new? The association between low birth weight and those factors is well-known and documented by several previous studies. It is better if you magnify or focus on a new and untouched variable.
  - ✓ After reviewing the available literature, we included potential risk factors for LBW and employed a new methodology of parameter estimation.
- Explain how you managed missing data, if any.
  - ✓ We only analysed complete cases in our analysis.
- Describe the purpose of using trace plots, kernel density plots, and ACF plots.
  - ✓ We explained what these plots indicate based on the output of results in the paper as per the reviewer's comment
- Have you considered a design-based analysis since the DHS survey employed a complex design?

- ✓ We applied the latent trait model that accounts for within and between-group variation while considering the multi-stage sampling design of the data.

- Present the overall estimation of LBW before presenting the regional variation.
  - ✓ We present the overall estimations in Tables 1 and 2 before presenting the regional estimation.
- In the result section, present only what you found, not how you found it.
  - ✓ We removed and corrected it accordingly
- Why did you prefer to use figures instead of tables? You may better organise and present several findings shown in Fig. 1, Fig. 2, etc
  - ✓ Since most covariates used in the study are continuous variables, scatter plots are better than tables to show the pattern.
- Overall, the result lacks a proper and clear description of statistical outputs in non-statistical explanations.
  - ✓ We have tried to correct this by considering the comment of the reviewer
- One of your findings is that the severity of LBW tends to decrease as the age of the mother increases. But is that always true among those mothers with an advanced age, which may be greater than 35 years? Have you conducted any sensitivity analyses?
  - ✓ We analysed the data and found that LBW severity decreases with age our finding also agrees with the findings of other authors
- Overall, the result lacks a proper and clear description of statistical outputs in non-statistical explanations.
  - ✓ We have made an effort to include all the comments accordingly.
- Apart from a simple comparison of findings, discussion should entail the clinical and practical implications of the findings.
  - ✓ We tried to incorporate the clinical and practical implications of the findings as per the reviewer's comment
- Recommendations are not specific, practical, or action-oriented.
  - ✓ We modified our recommendation based on our findings

Response to Reviewer 2

- Intrauterine is the main factor that causes LBW in developing countries in literature, can you explain this about your findings or your context and provide enough justification for why would it differ in the Ethiopian context? This is important because the connection with literature is vital.
    - ✓ Since we could not get enough evidence/ literature that indicated Intrauterine as the main factor of LBW we just removed it from the background.
- Additionally, using Bayesian estimation by itself is not enough gap to conduct research, the researchers must explore more to declare a clear gap as enough evidence on LBW is already available in Ethiopia.
    - ✓ We utilized the Bayesian method for parameter estimation as LBW is an ordinal qualitative variable. The MLE method can lead to biased and inaccurate probability coverage due to the presence of latent variables, hence, the Bayesian method was preferred.
- Your introduction section did not reflect the fact that many studies were conducted from different perspectives, you may need to dig more to show the gap in the literature.
    - ✓ We have tried to add studies conducted on rank likelihood by different authors as per the comment
- If you consider the Bayesian model as a novel (I reviewed many papers with Bayesian models), you need to present more methodological differences and provide enough evidence of how it is different from other traditional models to lay ground for readers.
    - ✓ We tried to compare the proposed model and the existing model (classical one) and showed the difference as per the comment of the reviewer
- Until in the method you did not provide evidence of source of data that is a big missing, please correct accordingly starting from abstract
    - ✓ We incorporated the source of our data as per the comment
- The data you used is indeed from DHS 2016, isn't this too old? There is a recent one in 2019. Or why you can not combine them to see the commutative effect or just use the recent Mini-DHS.
    - ✓ We used the EDHS 2016 because LBW data is not available in Mini-EDHS 2019.
- Methods are very limited as you said a golden approach, may need to include all method and material sections rather than present context and directly go to statistical

approaches e.g. data preparation, sampling, and handling missing…. generally, It is good to have a similar structure between the results and the method section.

  - ✓ We incorporated the comments accordingly

- What is the difference between Birth weights such as HBW NBW LBW VLBW ELBW and what does progress look like? (maybe in the table) because in Figure 1, I can see no difference among LBW, VLBW, and ELBW
  - ✓ In Table 1, we have tried to show the distribution of all levels of LBW and in Figure 1 we tried to show the pattern of LBW by region and age of mothers
- You need to improve the discussion as per the above comments
  - ✓ We improved the discussion as per the comments
- Conclusion must be more practical than tending to provide more general information.
  - ✓ We tried to make our conclusion more practical as per the comment
