# Supplementary material for: Bayesian rank likelihood-based estimation: An application to low birth weight in Ethiopia
Source: PLoS One. 2024 May 31;19(5):e0303637. doi: 10.1371/journal.pone.0303637 (PMC11142591; doi:10.1371/journal.pone.0303637)
Supplement: Attachment — Submitted filename: Response to reviewers.pdf [file pone.0303637.s003.pdf]

Responses to the reviewers' comments

First of all, we would like to say thank you for your comments.

Response to Reviewer 2

- The authors already published an article with similar findings from previous EDHS data(https://doi.org/10.5061/dryad.3j9kd51sg). This makes the reasoning for conducting the current study insufficient. What is the difference and how does the current recommendation show improvement from the previous one?
    - ✓ We would like to express our gratitude to the reviewer for reviewing our paper. However, we would like to clarify that we have not previously published any similar findings to the current study. We have made the data we used for our current study publicly available on the DRYAD repository, as per the guidelines of PLOS ONE. The data is accessible to everyone and can be used to gain further clarification about our study. We deposited a dataset to a public repository (DRYAD) and included the study's abstract and data description. The data repository team requested us to provide a title for the data that we deposited in DRYAD. We gave it a title that is almost identical to the title of the study and they published it online on the 28th of March 2024.
- The argument of a large sample covering a larger area of the country, even the study the authors published in March 2024 is larger and covered a more area of the country. so, how did this work out for the authors without making a clear link between this and the currently published one?
    - ✓ It is the data that we have used for the current study that was published in March 2024 not the full study. This is the link to the published dataset Rank likelihood-based estimation of low birth weight in Ethiopia (zenodo.org)
- Overall, the evidence on LBW is overly available already, and the current argument that because of using Bayesian Rank Likelihood Based Estimation needs to be carefully evaluated if it deserves research. The authors need to sequentially construct the flow of information including the most current evidence and show why it is important to research in the background/introduction.
    - ✓ In the background/introduction, we explained the importance of using the Bayesian rank likelihood method instead of the likelihood method. When dealing with ordered categorical variables, using the likelihood method can result in a biased estimator and inaccurate coverage probability. To address this

issue, we introduced the Bayesian method of parameter estimation for our current study.

- - For me, I can't make any difference between the published article (https://doi.org/10.5061/dryad.3j9kd51sg) and the currently under review manuscript.
    - ✓ https://doi.org/10.5061/dryad.3j9kd51sg) is the published data taken from the current study, it is not an article.
- It looks to me completely similar to what was published in March 2024 (same dependent variable, the same database, the same population but different articles). how that is possible. I would retake the revision in case I get somehow wrong.
    - ✓ We understand the confusion caused by the publication of the data before the study itself.

N.B: We deposited the dataset we used in the study in the public repository DRYAD as per the guideline of the PLOS ONE
