# Supplementary material for: Bayesian rank likelihood-based estimation: An application to low birth weight in Ethiopia
Source: PLoS One. 2024 May 31;19(5):e0303637. doi: 10.1371/journal.pone.0303637 (PMC11142591; doi:10.1371/journal.pone.0303637)
Supplement: Attachment — Submitted filename: PONE-D-23-37632.docx [file pone.0303637.s001.docx]

**Manuscript Number**: PONE-D-23-37632

**Article Type**: Research Article

**Full Title**: Bayesian Rank Likelihood Based Estimation: An Application to Low Birth Weight in Ethiopia

**General**

- Overall, a thorough review of the manuscript for grammatical issues is still required. Even within the abstract and introduction, there are multiple grammatical issues that make it difficult to read. Editing by a colleague or writing service with strong English skills is needed.

**Abstract:**

- It would be better if the abstract could be structured as follows: background, methods, results, and conclusion.
- As a justification for the study, you stated, "Most studies conducted in Ethiopia have utilized standard models employing maximum likelihood estimation with small sample sizes." But it could be better if you describe the limitations of those standard models and the advantage of Bayesian rank likelihood-based estimation over the standard models.
- The abstract should explain at least the data source, the study population, and the sample size.
- The abstract should be a stand-alone document. Some of your statements in the abstract are not standalone. For example, you stated, “The prevalence of low birth weight varies across different regions of the country, highlighting the need for targeted interventions in areas with a higher prevalence." This may need further clarification where the prevalence is high.
- The study revealed significant associations between birth weight and the mother's age, number of antenatal care visits, birth order, and BMI. What is new? The association between low birth weight and those factors is well known and documented by several previous studies. It is better if you magnify or focus on a new and untouched variable.
- As a recommendation, you said, “It is essential to implement community-based health promotion prioritizing antenatal care follow-up and maternal nutrition.” How? Where is the evidence-based intervention (i.e., community-based health promotion intervention)? Why not facility-based?
- Overall, in the abstract, I couldn’t find the answer to your study objective: to estimate parameters and provide a nationwide estimation of low birth weight and its risk factors in Ethiopia. At least you have to present the magnitude of LBW according to your estimation.

Background

- Please provide a comprehensive review of the epidemiology of LBW (magnitude, distribution, and determinants) in Ethiopia.
- In the background, you wrote, “According to [15], in the presence of ordinal variables, a maximum likelihood-based method yields estimates with substantial bias and inaccurate coverage probabilities.” Explain why these models are biased.
- In the background, a bit more detail is needed about the Bayesian rank likelihood method in the latent traits model.

**Methods and Materials**

- There are nine regions. Are you sure? Currently, there are twelve regional states and two chartered cities (Addis Ababa and Dire Dawa).
- Explain how you extract those variables from EDHS data sets.
- Explain how you managed missing data, if any.
- Describe the purpose of using trace plots, kernel density plots, and ACF plots.
- Have you considered a design-based analysis since the DHS survey employed a complex design?

**Results**

- When you present findings, start with a text description and refer the reader to any figure or table for details.
- In the result section, present only what you found, not how you found it.
- First, you need to present the characteristics of your study participants.
- Present the overall estimation of LBW before presenting the regional variation.
- Why did you prefer to use figures instead of tables? You may better organize and present several findings shown in Fig. 1, Fig. 2, etc.
- One of your findings is that the severity of LBW tends to decrease as the age of the mother increases. But is that always true among those mothers with an advanced age, which may be greater than 35 years? Have you conducted any sensitivity analyses?
- Overall, the result lacks a proper and clear description of statistical outputs in non-statistical explanations.

**Discussion**

- Apart from a simple comparison of findings, discussion should entail the clinical and practical implications of the findings.
- As part of the limitations of the study, you need to disclose the role of chance, bias, and confounding factors in your findings. And how do you minimize or control their effects?
- Recommendations are not specific, practical, or action-oriented. (i.e., targeted public health interventions, community-based health promotion activities, etc.)
